# Associations of the glutathione S-transferase P1 Ile105Val genetic polymorphism with gynecological cancer susceptibility: a meta-analysis

**DOI:** 10.18632/oncotarget.16764

**Published:** 2017-03-31

**Authors:** Erjiang Zhao, Kai Hu, Yan Zhao

**Affiliations:** ^1^ Affiliated Tumor Hospital of Zhengzhou University, Henan Tumor Hospital, Zhengzhou, China; ^2^ Henan Academy of Medical Sciences, Zhengzhou, Henan, China

**Keywords:** GSTP1, polymorphism, meta-analysis, cervical cancer, endometrial cancer

## Abstract

The association between the glutathione S-transferase P1 (GSTP1) Ile105Val polymorphism and gynecological cancer susceptibility has been evaluated in many studies. However, the results remain controversial. Thus, this meta-analysis, based on 10 published case-control studies, was designed to clarify the association of the GSTP1 Ile105Val polymorphism with gynecological cancer risk. Our results suggested that there was no significant association between the GSTP1 Ile105Val polymorphism and the risk of gynecological cancer in all genetic models (GG *vs*. AA: odds ratio [OR] = 1.41, 95% confidence interval [CI] = 0.75-2.26; AG *vs*. AA: OR = 1.13, 95% CI = 0.74-1.73; AG/GG *vs*. AA: OR = 1.17, 95% CI = 0.75-1.81; GG *vs*. AA/AG: OR = 1.38, 95% CI = 0.79-2.42). Similarly, in the subgroup analyses by cancer type, ethnicity, and smoking status, no significant association with any genetic model was observed. In conclusion, the results of our meta-analysis suggest that the GSTP1 Ile105Val polymorphism is not associated with the development of gynecological cancer.

## INTRODUCTION

Gynecologic malignancies are a major cause of cancer-related death in women worldwide. Cervical, endometrial, and ovarian cancer are the second, fourth, and fifth most common types of cancer, respectively, among North American women [1]. The exact molecular mechanisms underlying gynecologic malignancies are still poorly understood. However, as with other complex diseases, the influence of genetic factors, alone or in combination with local environmental factors, likely contributes to their development and progression. Especially, gene polymorphisms have been suggested as an important cause of the disparities in individual genetic susceptibility to gynecologic malignancies.

Glutathione S-transferases (GSTs) are a superfamily of phase II enzymes that are expressed in many tissues and are responsible for the metabolism of various xenobiotics and carcinogens by catalyzing the conjugation of glutathione to electrophilic compounds. They play a vital role in protecting the cells from oxidative damage and in modulating the induction of other enzymes and proteins in response to DNA damage; therefore, they are important for maintaining genomic integrity [2]. Numerous polymorphisms have been reported to occur in the genes encoding GSTs, which may reduce their efficiency and increase the risk of certain cancers. Accordingly, GSTs have been widely explored as risk biomarkers for various cancers, including gynecological cancers. Among these enzymes, the GST class π enzyme encoded by the GSTP1 gene is overexpressed in various tumor types and is a major enzyme involved in the inactivation of cigarette smoke carcinogens and other toxic constituents. A single nucleotide polymorphism in the GSTP1 gene at codon 105 (Ile105Val), which causes an amino acid substitution of isoleucine (Ile) by valine (Val), results in decreased enzymatic activity and lowers the ability to metabolize certain xenobiotics and carcinogens. Biochemical studies have indicated that the GSTP1/Val105 variant is 2-3 times less stable than the Ile105 variant [3] and may be associated with the risk of gynecological cancers.

In the past decade, several case-control studies about the relationship between the GSTP1 Ile105Val polymorphism and gynecological cancer susceptibility have been conducted. However, the results are still inconclusive, owing to the relatively small sample sizes of the studies, resulting in limited power to estimate the overall effects. Meta-analyses are powerful tools for summarizing inconclusive results from individual studies. Therefore, we performed the present meta-analysis using data from 10 previously published case-control studies [4–13] to more precisely assess the association between the GSTP1 Ile105Val polymorphism and gynecological cancer risk.

## RESULTS

### Eligible studies

**Figure 1:**
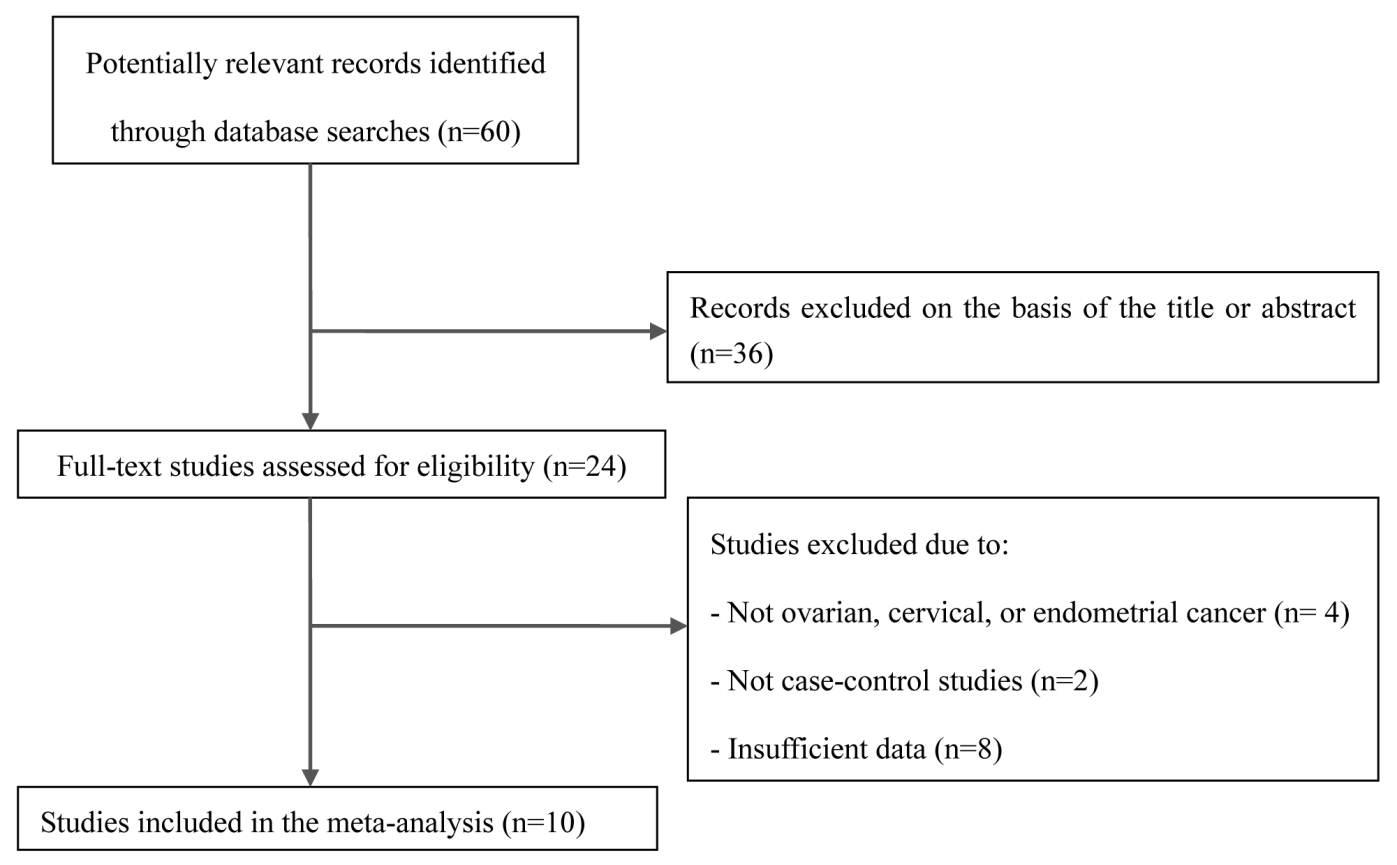
Flow chart of the selection of studies included in the current meta-analysis

As shown in Figure 1, 60 potentially eligible studies were retrieved through the initial search. After screening the articles as described above, 50 articles failed to meet the inclusion criteria and were hence excluded. Thus, 10 eligible articles, including 1339 cases and 2884 controls, were finally included in this meta-analysis [4–13]. The main characteristics of these studies are summarized in Table 1. The included studies included 4, 4, and 2 studies on ovarian cancer, cervical cancer, and endometrial cancer, respectively. Four studies each were conducted specifically in Asian and Caucasian populations. Data on the smoking status and GSTP1 genotypes were available in 2 studies. The minor allele frequency of the G allele among the controls in these 10 studies ranged from 0.04 to 0.38.

**Table 1:** Main characteristics of the studies included in this meta-analysis

First author [ref]	Year	Country	Ethnicity	Cancer type	Source of controls	Genotype distribution						P for HWE^a^	G
						Cases (n)			Controls (n)				
						AA	AG	GG	AA	AG	GG		
Spurdle [4]	2001	Australia	Caucasian	Ovarian cancer	PB	121	130	31	114	135	43	0.77	0.38
Jee [5]	2002	Korea	Asian	Cervical cancer	HB	220	108	14	472	207	28	0.38	0.19
Chan [6]	2005	China	Asian	Endometrial cancer	HB	87	86	7	130	62	8	0.86	0.20
Sobti [7]	2006	India	Asian	Cervical cancer	HB	31	68	4	32	68	3	0.00	0.36
Morari [8]	2006	Brazil	Brazilian	Ovarian cancer	PB	33	26	10	98	94	30	0.33	0.35
Delort [9]	2008	France	Caucasian	Ovarian cancer	PB	26	20	5	916	80	4	0.12	0.04
Palma [10]	2010	Italy	Caucasian	Cervical cancer	HB	40	35	6	55	53	3	0.02	0.27
Kiran [11]	2010	Turkish	Asian	Cervical cancer	HB	27	15	4	22	26	2	0.09	0.30
Oliveira [12]	2012	Brazil	Brazilian	Ovarian cancer	HB	76	42	14	60	59	13	0.79	0.32
Ozerkan [13]	2013	Turkey	Caucasian	Endometrial cancer	HB	27	23	3	28	30	9	0.83	0.36

^a^In the controls.

HB: hospital-based; PB: population-based; HWE, Hardy-Weinberg equilibrium.

### Meta-analysis

**Figure 2:**
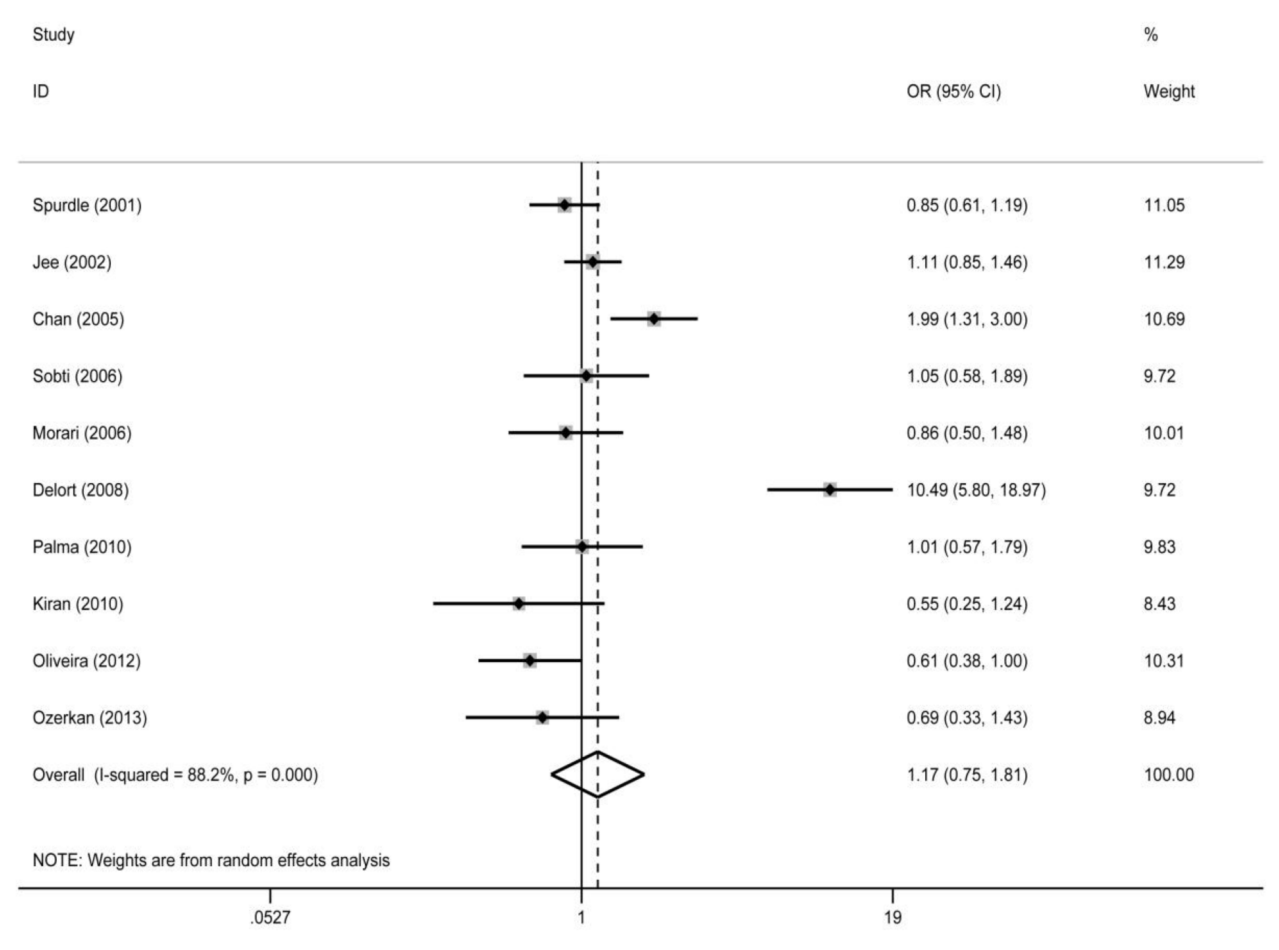
Forest plot for the association between the glutathione S-transferase P1 (GSTP1) Ile105Val polymorphism and gynecologic cancer risk for the AG/GG genotype compared with the AA genotype OR, odds ratio; CI, confidence interval.

For the overall study population, our study did not detect a significant association between the GSTP1 Ile105Val polymorphism and gynecological cancer risk in any of the genetic models (GG vs. AA: OR = 1.41, 95% CI = 0.75-2.26; AG vs. AA: OR = 1.13, 95% CI = 0.74-1.73; AG/GG vs. AA: OR = 1.17, 95% CI = 0.75-1.81 (Figure 2); GG vs. AA/AG: OR =1.38, 95% CI = 0.79-2.42). In the subgroup analysis by cancer type, the GSTP1 Ile105Val polymorphism did not associate with the risk of ovarian, cervical, or endometrial cancer. In addition, we did not find an association in the stratified analyses by ethnicity or smoking status (Table 2).

**Table 2:** Summary odds ratios (ORs) and 95% confidence intervals (CIs) for the associations between the glutathione S-transferase P1 (GSTP1) Ile105Val polymorphism and gynecologic cancer risk

Subgroups	*n*	GG *vs*. AA		AG *vs*. AA		AG/GG *vs*. AA		GG *vs*. AA/AG	
		OR (95% CI)	*I*^2^	OR (95% CI)	*I*^2^	OR (95% CI)	*I*^2^	OR (95% CI)	*I*^2^
All studies	10	1.41 (0.75-2.66)	75.1%	1.13 (0.74-1.73)	86.0%	1.17 (0.75-1.81)	88.2%	1.38 (0.79-2.42)	69.6%
Cancer type									
Cervical	4	1.32 (0.78-2.23)	0.0%	1.01 (0.80-1.26)	20.2%	1.03 (0.83-1.29)	0.0%	1.34 (0.80-2.24)	0.0%
Ovarian	4	1.96 (0.52-7.43)	90.5%	1.37 (0.48-3.89)	94.2%	1.45 (0.48-4.37)	95.3%	1.88 (0.61-5.80)	87.9%
Endometrial	2	0.78 (0.35-1.77)	54.7%	1.35 (0.53-3.44)	78.6%	1.22 (0.44-3.43)	83.7%	0.67 (0.30-1.51)	10.6%
Ethnicity									
Caucasian	4	2.24 (0.32-15.47)	91.6%	1.54 (0.54-4.45)	93.1%	1.58 (0.49-5.07)	94.8%	2.07 (0.38-11.25)	89.6%
Asian	4	1.19 (0.72-1.97)	0.0%	1.13 (0.70-1.83)	73.9%	1.16 (0.75-1.77)	69.1%	1.12 (0.69-1.84)	0.0%
Smoking status									
Smoker	2	2.72 (0.28-26.86)	0.0%	1.31 (0.59-2.89)	0.0%	1.40 (0.64-3.08)	0.0%	2.31 (0.24-21.97)	0.0%
Non-smoker	2	1.80 (0.50-6.51)	0.0%	1.04 (0.58-1.86)	0.0%	1.10 (0.62-1.95)	0.0%	1.77 (0.52-6.02)	0.0%

### Publication bias

**Figure 3:**
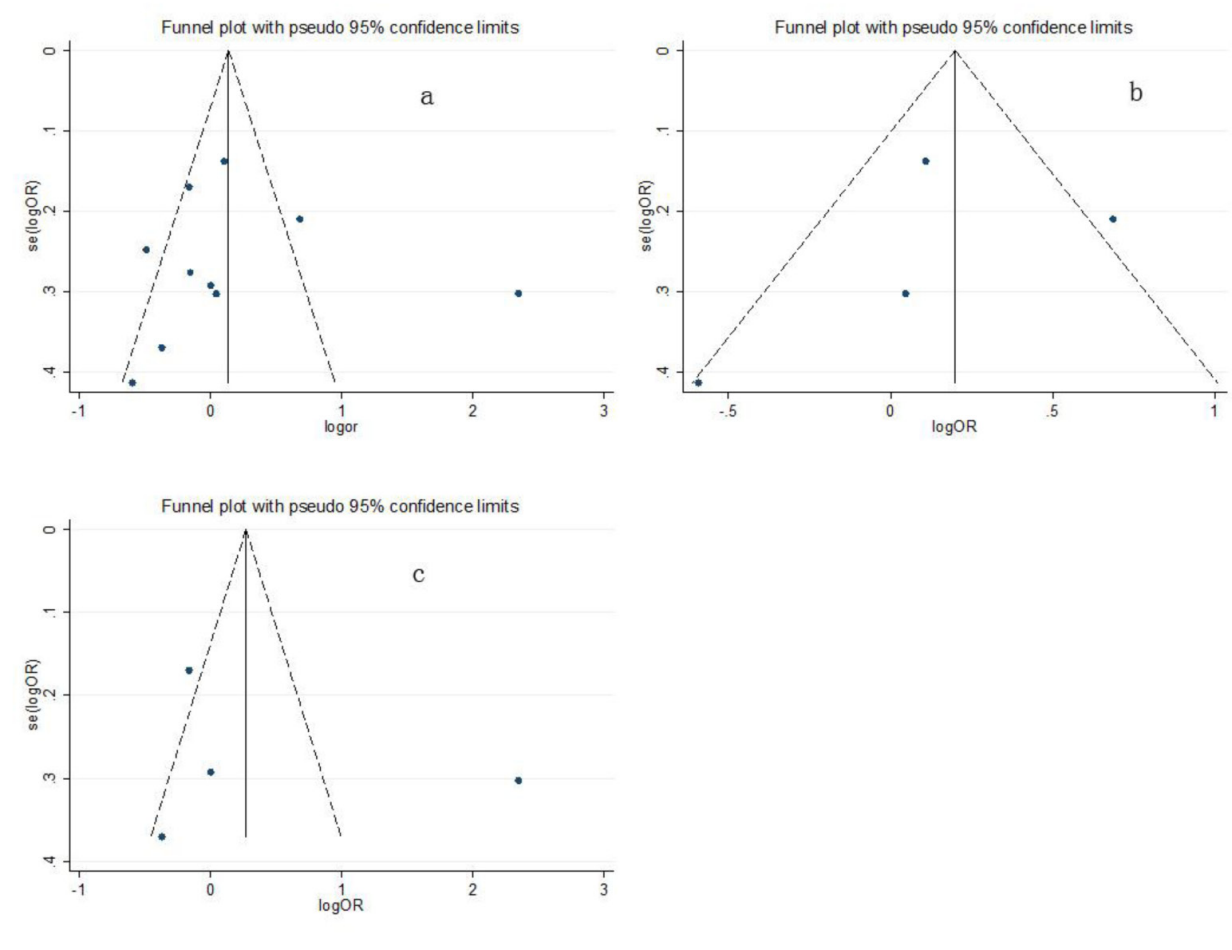
Funnel plots of the associations between the glutathione S-transferase P1 (GSTP1) Ile105Val polymorphism and gynecologic cancer risk for the AG/GG genotype compared with the AA genotype in (a) the overall population, (b) Asian subjects, and (c) Caucasian subjects

Funnel plots were created and Egger's test was performed to assess the publication bias of the included studies. The funnel plots did not show obvious asymmetry in the overall population. The results of Egger's linear regression test confirmed the funnel plot symmetry (GG vs. AA: t = 1.66, *P* = 0.134; GA vs. AA: t = 0.51, *P* = 0.623; GA/GG vs. AA: t = 0.14, P = 0.893 (Figure 3); GG vs. AA/GA: t = 1.71, *P* = 0.126).

## DISCUSSION

To the best of our knowledge, this is the first meta-analysis to assess the association between the GSTP1 Ile105Val polymorphism and the risk of gynecological cancer, a controversial topic. The results of our meta-analysis did not indicate any significant association between the GSTP1 Ile105Val polymorphism and the risk of gynecological cancer, both in the overall study population and in the stratified subgroup analyses.

The GSTP1 Ile105Val polymorphism has been widely investigated as a risk biomarker for various cancers, such as chronic myeloid leukemia [14], esophageal cancer [15], and bladder cancer [16], among others. However, a previous meta-analysis of genetic data showed that the GSTP1 Ile105Val polymorphism was not associated with the risk of ovarian cancer [17], and these results are in agreement with those of the present meta-analysis. Of note, our meta-analysis included one new study that was not included in the previous meta-analysis. Therefore, the present analysis obtained stronger statistical power. Meanwhile, unlike in the present study, the previous meta-analysis did not explore the associations between the GSTP1 Ile105Val polymorphism and the risks of cervical and endometrial cancer. Similar to for ovarian cancer, however, the present meta-analysis did not find a significant association between the GSTP1 Ile105Val polymorphism and cervical or endometrial cancer risk.

As has been well established, tobacco smoking is a significant risk factor for many cancer types [18]. GSTP1 has been hypothesized to be involved in metabolizing the various carcinogens present in cigarette smoke, including, among others, polycyclic aromatic hydrocarbons, and a lack of GSTP1 activity might hence ultimately increase the burden of these carcinogens. Interestingly, in our study, no interaction between GSTP1 and gynecological cancer was detected in either ever-smokers or non-smokers, suggesting that cigarette smoking might not significantly modify the association between the GSTP1 Ile105Val polymorphism and gynecological cancer susceptibility. However, the results should be interpreted with caution due to the relatively small sample size in the subgroup analysis according to smoking status.

Meanwhile, many genes likely influence the polycyclic aromatic hydrocarbon metabolism. Therefore, gene-gene interactions are likely more appropriate as risk factors of gynecological cancer than a single gene. Palma et al. [10] reported that, while the GST gene alone was not associated with gynecological cancer, a combination of the GSTM1 null, GSTT1 null, and GSTP1 AA genotypes associated with an increased risk of gynecological cancer.

Although our meta-analysis is robust, several limitations should be acknowledged. First, in the subgroup analyses by cancer type, ethnicity, and smoking status, the relatively small sample sizes may have led to insufficient power to detect the real relationships. Second, the data of each included study were based on unadjusted estimates, and a more precise analysis could have been performed if the original data were available. Third, gene-gene interactions might play an important role in the development of gynecological cancers. However, we could not carry out analyses on these interactions due to insufficient data.

In summary, the results of the present meta-analysis suggest that the GSTP1 Ile105Val polymorphism is not associated with the development of gynecological cancer. Further well-designed studies with larger sample sizes are required to confirm this conclusion.

## MATERIALS AND METHODS

### Search strategy

Systematic searches were performed in the PubMed and Elsevier databases (until December 2015) using combinations of the following search terms: ‘GSTP1,’ ‘glutathione S-transferase P1,’ ‘polymorphism,’ ‘gynecologic malignancies,’ ‘ovarian cancer,’ ‘cervical cancer,’ and ‘endometrial cancer.’ Moreover, additional eligible articles were identified through scanning of the references cited in the retrieved articles. We assessed the titles and abstracts of the retrieved articles to exclude any irrelevant studies. Additionally, the remaining full-text articles were thoroughly scrutinized to determine whether they were suitable for the final analysis.

### Inclusion and exclusion criteria

The inclusion criteria were as follows: (1) case-control studies that were conducted to assess the association between the GSTP1 Ile105Val polymorphism and gynecological cancer risk and (2) studies with sufficient data for estimating odds ratios (ORs) and their 95% confidence intervals (CIs). Gynecologic malignancies were defined as cervical cancer, endometrial cancer, and ovarian cancer. In cases of multiple publications using the same or overlapping data, we chose the one with the largest number of subjects. The exclusion criteria were as follows: (1) reviews and comments; (2) studies with duplicate data; and (3) articles with no available information on the genotype frequency.

### Data extraction

The data were obtained according to a standardized data extraction form. In order to minimize the risk of bias, two investigators extracted the following data from each study independently: the first author's name, year of publication, country, sources of controls (hospital-based or population-based), ethnicity (Asian or Caucasian), cancer type (ovarian, cervical, or endometrial cancer), smoking status (smokers or nonsmokers), numbers of different genotypes in the case and control groups (AA, AG, and GG genotypes), the Hardy-Weinberg equilibrium of the controls, and the minor allele frequency in the controls.

### Statistical analysis

The association between the GSTP1 Ile105Val polymorphism and the risk of gynecological cancer was estimated by using ORs with their corresponding 95% CIs. Pooled ORs were obtained for comparisons of heterozygotes (GA vs. AA), homozygotes (GG vs. AA), the dominant model (AG/GG vs. AA), and the recessive model (GG vs. AA/AG). Moreover, the studies were stratified and analyzed by ethnicity, cancer type, and smoking status. The Cochrane Q statistics test and I-squared (I2) metric were used to test heterogeneity. When the I2 for heterogeneity was >50%, the fixed effects model was used. Otherwise, the random effects model was applied (DerSimonian and Laird method) [19]. Funnel plots and Egger's linear regression test [20] were used to identify potential publication bias. All statistical analyses were performed using STATA 11.0 software (STATA Corp., College Station, TX, USA), with two-sided P values < 0.05 considered significant.

## References

[1] Siegel R, Ward E, Brawley O, Jemal A (2011). Cancer statistics, 2011: the impact of eliminating socioeconomic and racial disparities on premature cancer deaths. CA Cancer J Clin.

[2] Hayes JD, Flanagan JU, Jowsey IR (2005). Glutathione transferases. Annu Rev Pharmacol Toxicol.

[3] Johansson AS, Stenberg G, Widersten M, Mannervik B (1998). Structure-activity relationships and thermal stability of human glutathione transferase P1-1 governed by the H-site residue 105. J Mol Biol.

[4] Spurdle AB, Webb PM, Purdie DM, Chen X, Green A, Chenevix-Trench G (2001). Polymorphisms at the glutathione S-transferase GSTM1, GSTT1 and GSTP1 loci: risk of ovarian cancer by histological subtype. Carcinogenesis.

[5] Jee SH, Lee JE, Kim S, Kim JH, Um SJ, Lee SJ, Namkoong SE, Park JS (2002). GSTP1 polymorphism, cigarette smoking and cervical cancer risk in Korean women. Yonsei Med J.

[6] Chan QK, Khoo US, Ngan HY, Yang CQ, Xue WC, Chan KY, Chiu PM, Ip PP, Cheung AN (2005). Single nucleotide polymorphism of pi-class glutathione s-transferase and susceptibility to endometrial carcinoma. Clin Cancer Res.

[7] Sobti RC, Kaur S, Kaur P, Singh J, Gupta I, Jain V, Nakahara A (2006). Interaction of passive smoking with GST (GSTM1, GSTT1, and GSTP1) genotypes in the risk of cervical cancer in India. Cancer Genet Cytogenet.

[8] Morari EC, Lima AB, Bufalo NE, Leite JL, Granja F, Ward LS (2006). Role of glutathione-S-transferase and codon 72 of P53 genotypes in epithelial ovarian cancer patients. J Cancer Res Clin Oncol.

[9] Delort L, Chalabi N, Satih S, Rabiau N, Kwiatkowski F, Bignon YJ, Bernard-Gallon DJ (2008). Association between genetic polymorphisms and ovarian cancer risk. Anticancer Res.

[10] Palma S, Novelli F, Padua L, Venuti A, Prignano G, Mariani L, Cozzi R, Tirindelli D, Testa A (2010). Interaction between glutathione-S-transferase polymorphisms, smoking habit, and HPV infection in cervical cancer risk. J Cancer Res Clin Oncol.

[11] Kiran B, Karkucak M, Ozan H, Yakut T, Ozerkan K, Sag S, Ture M (2010). GST (GSTM1, GSTT1, and GSTP1) polymorphisms in the genetic susceptibility of Turkish patients to cervical cancer. J Gynecol Oncol.

[12] Oliveira C, Lourenço GJ, Sagarra RA, Derchain SF, Segalla JG, Lima CS (2012). Polymorphisms of glutathione S-transferase Mu 1 (GSTM1), Theta 1 (GSTT1), and Pi 1 (GSTP1) genes and epithelial ovarian cancer risk. Dis Markers.

[13] Ozerkan K, Atalay MA, Yakut T, Doster Y, Yilmaz E, Karkucak M (2013). Polymorphisms of glutathione-s-transferase M1, T1, and P1 genes in endometrial carcinoma. Eur J Gynaecol Oncol.

[14] Sailaja K, Surekha D, Rao DN, Rao DR, Vishnupriya S (2010). Association of the GSTP1 gene (Ile105Val) polymorphism with chronic myeloid leukemia. Asian Pac J Cancer Prev.

[15] Song Y, Du Y, Zhou Q, Ma J, Yu J, Tao X, Zhang F (2014). Association of GSTP1 Ile105Val polymorphism with risk of esophageal cancer: a meta-analysis of 21 case-control studies. Int J Clin Exp Med.

[16] Wang Z, Xue L, Chong T, Li H, Chen H, Wang Z (2013). Quantitative assessment of the association between glutathione S-transferase P1 Ile105Val polymorphism and bladder cancer risk. Tumour Biol.

[17] Economopoulos KP, Sergentanis TN, Vlahos NF (2010). Glutathione S-transferase M1, T1, and P1 polymorphisms and ovarian cancer risk: a meta-analysis. Int J Gynecol Cancer.

[18] Raimondi S, Paracchini V, Autrup H, Barros-Dios JM, Benhamou S, Boffetta P, Cote ML, Dialyna IA, Dolzan V, Filiberti R, Garte S, Hirvonen A, Husgafvel-Pursiainen K (2006). Meta- and pooled analysis of GSTT1 and lung cancer: a HuGE-GSEC review. Am J Epidemiol.

[19] Mantel N, Haenszel W (1959). Statistical aspects of the analysis of data from retrospective studies of disease. J Natl Cancer Inst.

[20] Egger M, Davey Smith G, Schneider M, Minder C (1997). Bias in meta-analysis detected by a simple, graphical test. BMJ.

